# Disease Outbreak Surge Response: How a Singapore Tertiary Hospital Converted a Multi-story Carpark Into a Flu Screening Area to Respond to the COVID-19 Pandemic

**DOI:** 10.1017/dmp.2020.249

**Published:** 2020-07-14

**Authors:** Jeevan Raaj Thangayah, Kenneth Boon Kiat Tan, Chin Siah Lim, Tzay-Ping Fua

**Affiliations:** Singapore General Hospital, Department of Emergency Medicine, Singapore

**Keywords:** Emergency medicine, emerging infectious diseases, infectious disease outbreak, surge capacity

## Abstract

Coronavirus disease 2019 (COVID-19), first documented in December 2019, was declared a public health emergency by the World Health Organization (WHO) on January 30, 2020 (https://www.who.int/westernpacific/emergencies/covid-19). The disease, caused by the severe acute respiratory syndrome coronavirus 2 (SARS-CoV-2) virus, has affected more than 9 million people and contributed to at least 490,000 deaths globally as of June 2020, with numbers on the rise (https://www.worldometers.info/coronavirus/#countries).

Increased numbers of patients seeking medical attention during disease outbreaks can overwhelm healthcare facilities, hence requiring an equivalent response from healthcare services. Surge capacity is a concept that has not only been defined as the “ability to respond to a sudden increase in patient care demands” (Hick et al., *Disaster Med Public Health Prep*. 2008;2:S51-S57) but also to “effectively and rapidly expand capacity” (Watson et al., *Milbank Q*. 2013;91(1):78-122).

This narrative review discusses how Singapore’s largest tertiary hospital has encapsulated the elements of surge capability and transformed a peacetime multi-story carpark into a flu screening area in response to the COVID-19 disease outbreak.

The first case of coronavirus disease 2019 (COVID-19) in Singapore was reported on January 23, 2020, and as of June 2020, over 42,000 cases have been reported to local authorities.^1^ As part of Singapore’s national strategy for a pandemic response, the DORSCON (Disease Outbreak Response System Condition) surveillance level was raised from Green to Orange on February 7, 2020.

DORSCON is a color-coded framework developed by the Ministry of Health (MOH), Singapore, as a means of characterizing a disease outbreak (DO) in terms of threat of infection to the general public, the condition of the DO overseas, and the impact of the DO on the local community. The color-coding ranges from green, which corresponds to a negligible public health impact, to red, referring to high public health impact (refer to the Appendix).

In modern-day Singapore, our experiences with pandemics, among several other outbreaks, include severe acute respiratory syndrome (SARS) in 2003 and H1N1 in 2009 with thirty-three^2^ and twenty-one^3^ fatalities, respectively.

As part of the nation’s infectious disease outbreak preparedness, the Ministry of Health (MOH), Singapore, mandated that all public hospitals in Singapore have a flu screening area (FSA). Accordingly, at Singapore General Hospital (SGH), we designated a multi-story carpark (MSCP) to be used as our FSA during a pandemic.

## Feasibility of the MSCP to Be Converted to an FSA

Planning for the MSCP to be converted to an FSA was initiated as early as 2013 with the acquisition of buy-in and approval from management committees and relevant stakeholders. In 2015, internal validation of the infrastructure and workflows through simulation took place and is a continual process as developments in processes are made. Our hospital’s Preparedness and Response (P&R) unit is heavily involved in this process. In addition, the Department of Infection Prevention and Epidemiology (IPE) ensures that all workflows and processes strictly adhere to infection control standards.

**FIGURE 1:**
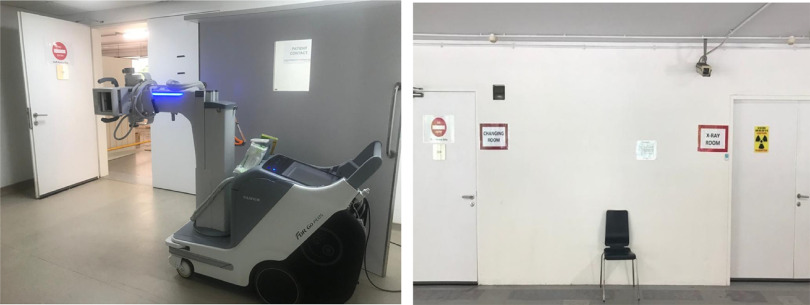
Radiology Facilities in the Flu Screening Area (FSA).

**FIGURE 2:**
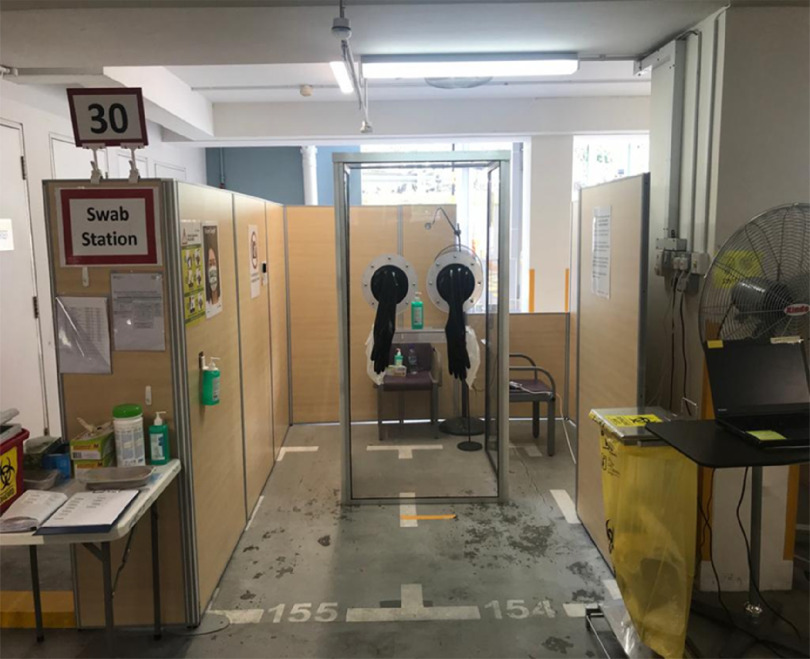
Swab Booth.

**FIGURE 3:**
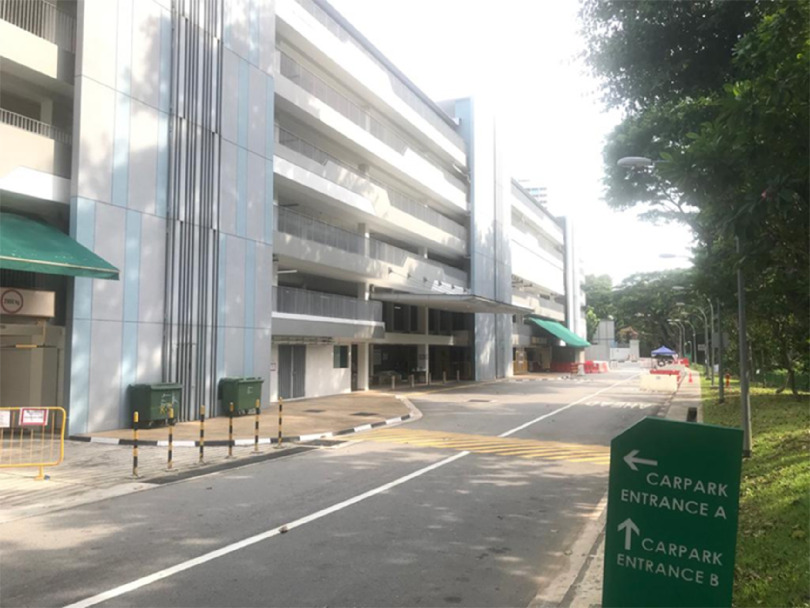
Multi-story Carpark (MCSP).

**FIGURE 4:**
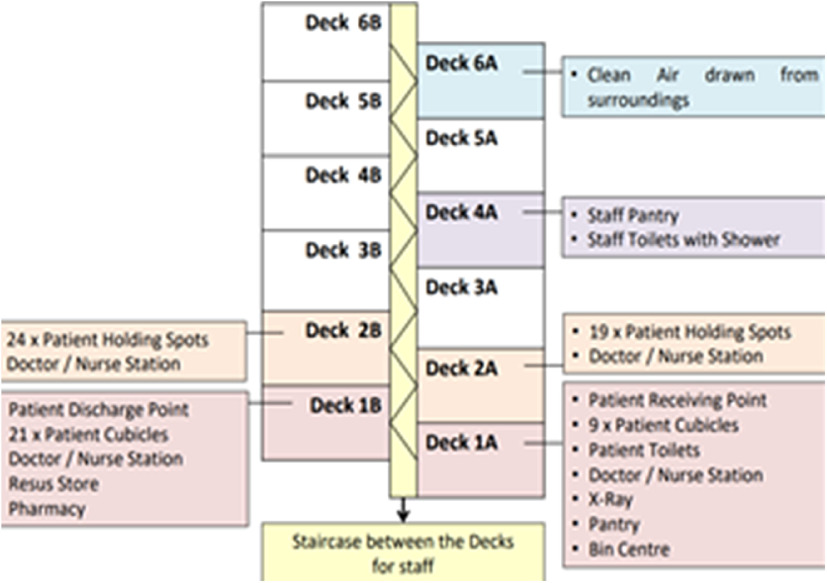
MSCP Deck Layout as a Flu Screening Area (FSA).

**FIGURE 5:**
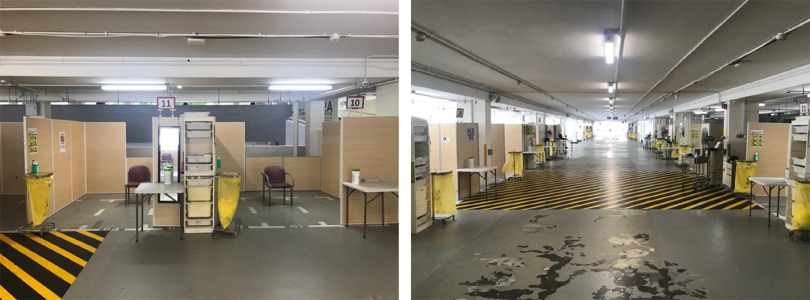
Cubicles Replete With Chairs, IT Equipment, PPE, Disinfectants, and Waste Disposal.

**FIGURE 6:**
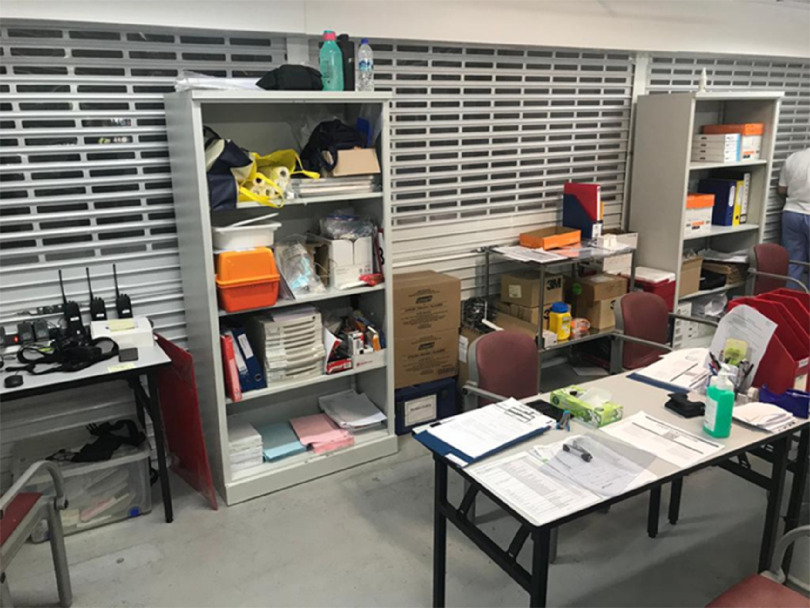
Air-Conditioned, Shuttered Staff Clinical Work Areas.

**FIGURE 7:**
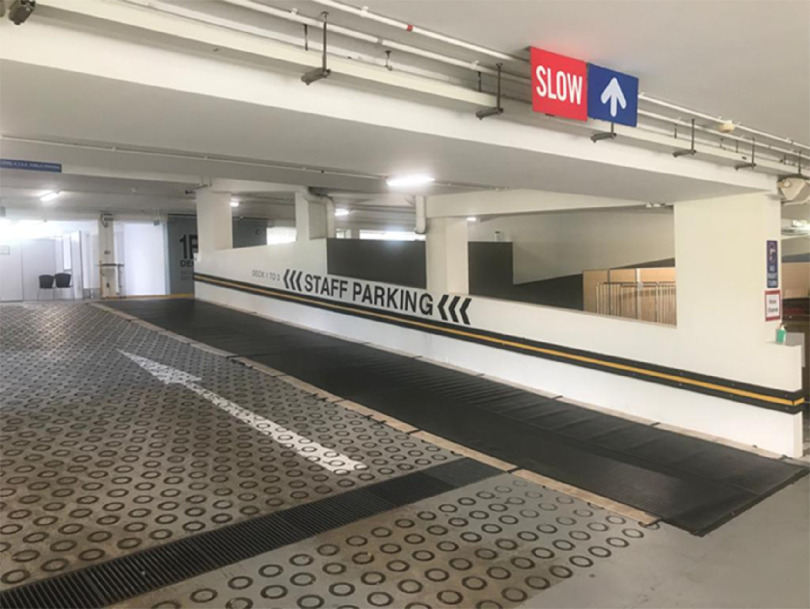
Modified Ramp.

The MSCP is a permanent structure that operates as a parking facility during peacetime for staff and members of the public. A turnaround time of 72 h is required to convert the parking facility into an FSA, replete with information technology (IT) support, resuscitation equipment, and patient care cubicles. There is an on-site X-ray room as shown in Figure 1. A dedicated swab station is also specially set up for the current outbreak (Figure 2). The MSCP (Figure 3) is some 800 m away from the main hospital building, thus serving as an ideal location to screen stable, suspect infectious patients away from the main hospital building. The site is easily accessed by means of main roads for ease of transporting patients and for code blue teams to arrive on scene from the main hospital building where the emergency department (ED) is located. It has a large surface area spanning 12 decks (Figure 4), which can be converted to clinical work areas as shown in Figures 5 and 6. It is also well ventilated to minimize aerosol transmission, compared with enclosed spaces. A centrally located staircase was retrofitted to the infrastructure for staff use only during DO response, to minimize contact with suspect cases. Patients’ movements will be by means of car ramps (Figure 7) to minimize crossing paths with healthcare staff. The FSA would serve as an extension of the Department of Emergency Medicine’s (DEM) response to an infectious DO.

To maintain currency and sustainability, regular table top and physical exercises are conducted for staff. These staff include, but not limited to, doctors, nurses, senior management, and ancillary staff (eg., radiographers, pharmacists, housekeepers, and security officers). P&R and IPE play important roles in these exercises to ensure processes and workflows are safe and feasible.

## Transforming the MSCP Into an FSA: Encompassing the Elements of Surge Capacity

There are 4 tenets of surge capacity that have been well described in the literature, namely, staff, stuff, structure, and systems.^4^ In addition to augmenting staff during DO, ensuring the availability of comprehensive facilities, relevant equipment, and logistics are crucial to meet the increased healthcare demand. Strong support from policy-makers and senior hospital management is required to deliver successful patient care and achieve better overall outcomes. As part of SGH’s surge capability, a multi-disciplinary approach has been key to the successful transformation of the MSCP into an FSA.

The multi-disciplinary teams include medical and nursing staff from various departments in the hospital, as well as from other divisions and institutions within SGH’s campus. Support staff include, but are not limited to, security personnel, IT support, facilities management, general services such as ambulance drivers, and housekeeping. During its operational period, there was a total of 10 teams functioning at the FSA, with each team doing a 2-wk stint. Each team had between 3 and 10 doctors and 13 and 15 nurses coming together from various disciplines throughout the hospital. There was 1 shift daily from 8 am to 8 pm, with the teams working alternate days. The variation in the number of staff was due to the constantly changing demands at the FSA during its operational period.

## Workflow During COVID-19 Outbreak

On March 20, 2020, SGH was tasked by the MOH to activate the FSA, given the escalating situation of COVID-19 in Singapore. Once operationally ready, the FSA was seeing clinically stable suspect COVID-19 patients who are referred from primary healthcare settings and walk-ins to the main ED diverted to the FSA to avoid overcrowding in the main ED.

**FIGURE 8:**
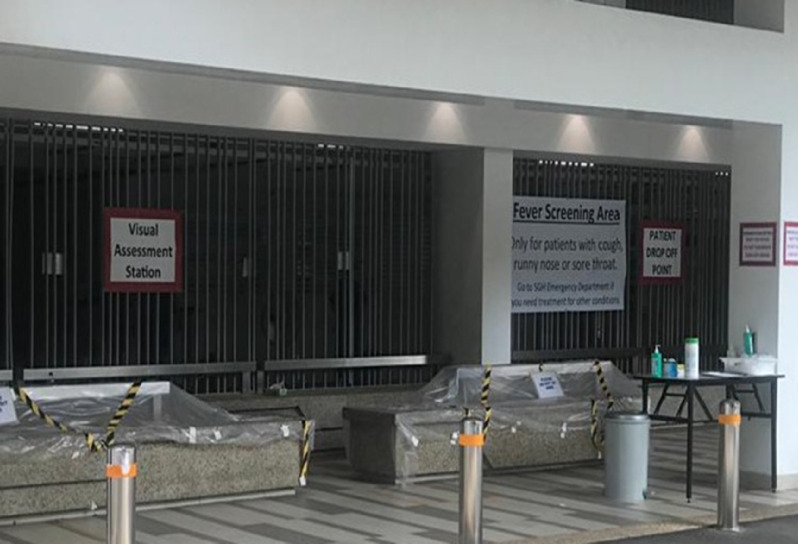
Visual Assessment Station (VAS).

**FIGURE 9:**
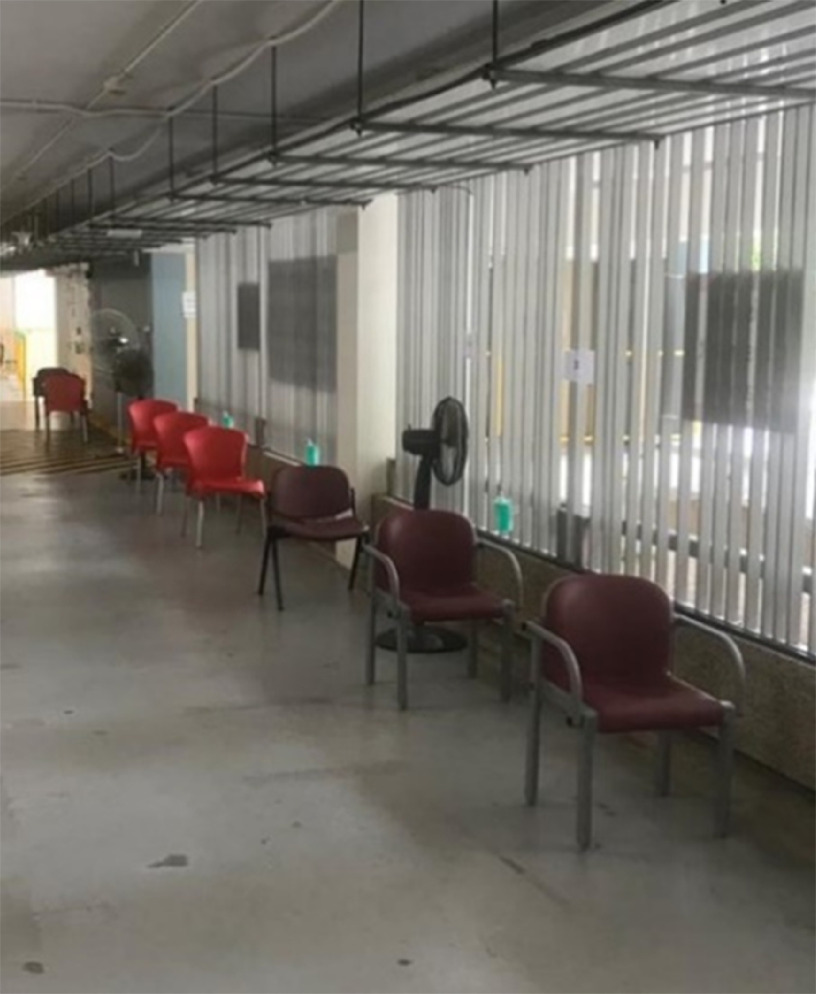
Chairs Placed 1 Meter Apart for Patients Waiting to be Seen.

Patients are first seen at the Visual Assessment Station (VAS), where they are screened by trained nurses and assessed for the suitability to be seen at the FSA (Figure 8). Patients requiring medical attention for symptoms other than mild respiratory tract symptoms with fever are diverted to the main ED. In the event of an increase in the number of patients waiting to be screened, chairs at the VAS are placed apart from each other according to IPE recommendations for patients to sit while awaiting their turn (Figure 9).

**FIGURE 10:**
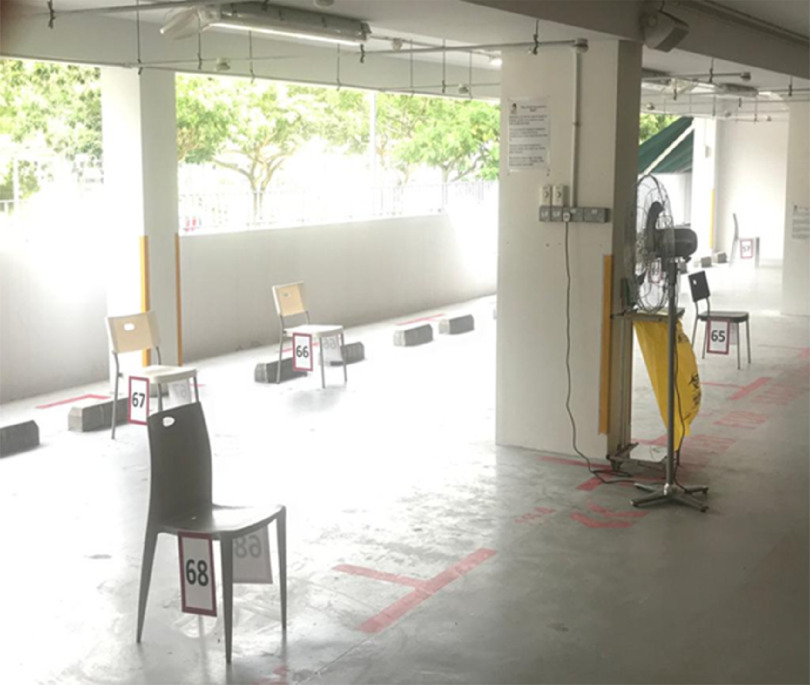
Waiting Areas for Patients.

Thereafter, patients will be seen by a doctor in full personal protective equipment (PPE) in a cubicle and sent to the swab booth for specimen taking. Full PPE comprises N95 mask; face shield or goggles; long-sleeved, below-knee length gown; and gloves. The swab is performed at a dedicated swab station, which comprises a 3-walled booth made of polycarbonate with attached neoprene gloves. This structure does not require doctors to wear PPE gowns, hence avoiding their unnecessary use. Patients will then be directed to the chest X-ray (CXR) room that is specially set up at the FSA. While awaiting the formal CXR report, patients will be directed to be seated at waiting areas (Figure 10).

After the swab and CXR are performed, patients fit for discharge will be directed to the pharmacy, and finally, they will exit the FSA, which is opposite to the pharmacy. This is the dedicated patients’ exit point out of the FSA, which is on a separate deck from the patients’ entrance point. After each patient is seen, the consultation cubicles are cleaned by environmental services staff who have been trained in infection control. They strictly adhere to cleaning protocols set out by the IPE.

**FIGURE 11:**
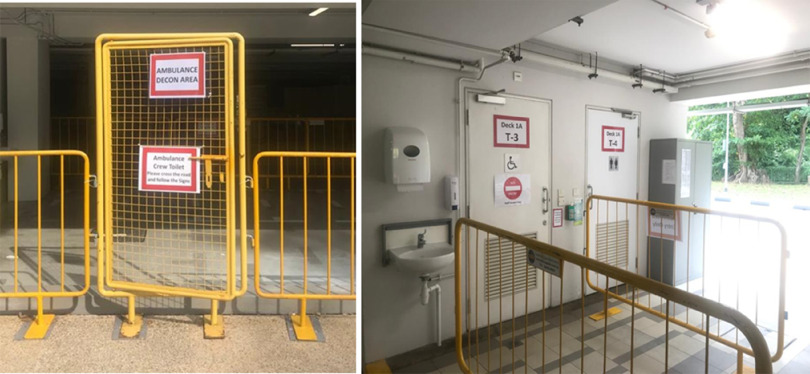
Ambulance Crew Decontamination Areas.

To avoid unnecessary contact with suspect patients, there are dedicated staff entrances and staircases. The ramps between each level of the FSA are modified with platforms to allow easy transport of trolleys and equipment. Ambulance crew who have transported suspect cases also have dedicated decontamination areas (Figure 11). Dedicated staff rest areas and staff toilets are located on the upper floors of the MSCP with a buffer of 2 floors between clinical areas and staff rest areas for further expansion if needed. Staff rest areas and nursing stations are air conditioned, and the windows have shutters.

The FSA is supported by Wi-Fi throughout the facility. In addition, the IT system used at the FSA is the same as the one used in the main ED and throughout the hospital. Hence, all laboratory and radiological investigation results are received electronically by means of the same IT system at the FSA.

On average, SGH ED sees a total of 340 patients daily.^5^ During its operational period from March 20, 2020, to May 13, 2020, the FSA saw between 5 and 15% of the monthly total ED patient load. According to our institutional data, during the FSA’s operational period, an average of 1 patient per day was transferred from the FSA to the main ED after an initial FSA consultation, for evaluation by emergency physicians or other specialists’ input.

Patients registered at the FSA waited for an average of 34 min to be evaluated by a doctor and between 2 and 4 h to reach a disposition. None had refused testing and no adverse events occurred. Patients were swabbed and discharged from the FSA with a follow-up call being made the next day by the IPE team. Also, they could access the result of the swab test by means of a smartphone app if they desired. All patients seen at the FSA were given at least a 5-d medical leave and were required, by law, to stay home for that whole duration. Patients whose swab test results returned positive were brought to the hospital from their homes by means of designated ambulances, to be admitted to isolation wards in hospitals across Singapore.

## CONCLUSION

To our knowledge, this is the only permanent structure in a Singapore hospital campus, with a nonmedical function during peacetime that can be converted to an FSA in times of a pandemic when patient load overwhelms existing resources. EDs should plan for a separate area or facility to deal with infectious disease surge to minimize the impact on daily workload. The development of the MSCP into an FSA serves its purpose as the physical “structure” component of our surge capability. An amalgamation of processes, dedication, and multidisciplinary commitment have enabled the success of this endeavor during this COVID-19 outbreak.

## Supplementary material

For supplementary material accompanying this paper visit http://dx.doi.org/10.1017/dmp.2020.249. click here to view supplementary material
