# Supplementary material for: Disease Outbreak Surge Response: How a Singapore Tertiary Hospital Converted a Multi-story Carpark Into a Flu Screening Area to Respond to the COVID-19 Pandemic
Source: Disaster Med Public Health Prep. 2020 Jul 14:1–6. doi: 10.1017/dmp.2020.249 (PMC7426610; doi:10.1017/dmp.2020.249)
Supplement: Supplementary file 1 [file S1935789320002499sup001.docx]

**Figures:**


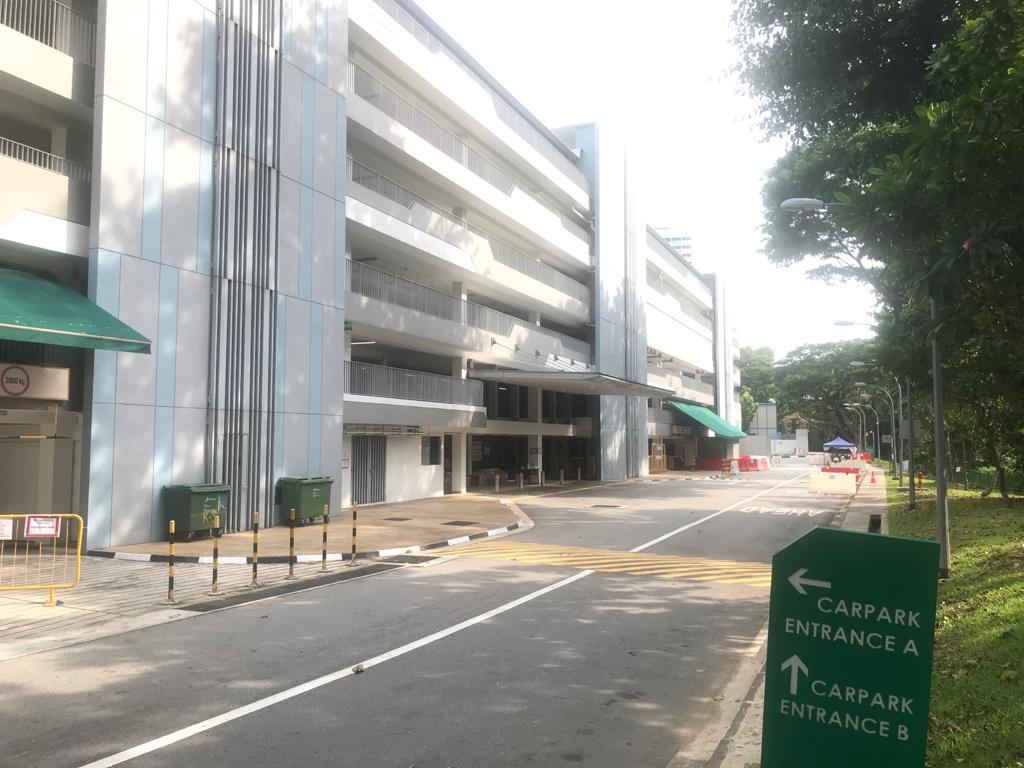

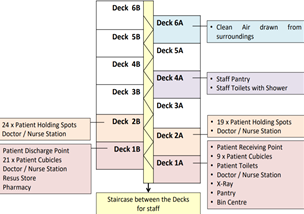


*Fig 4*. MSCP deck layout as a Flu Screening Area (FSA).

*Fig 3*. Multi-story carpark (MSCP).


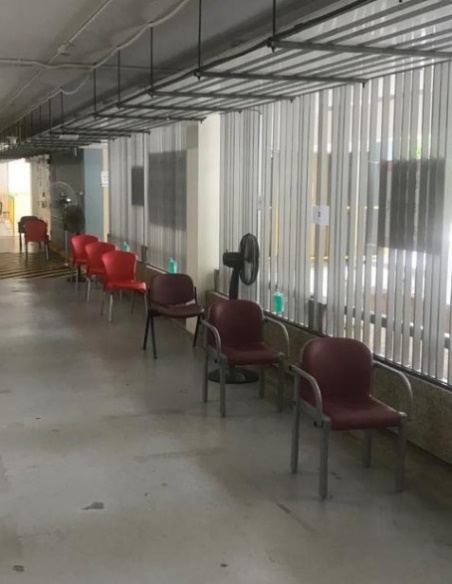

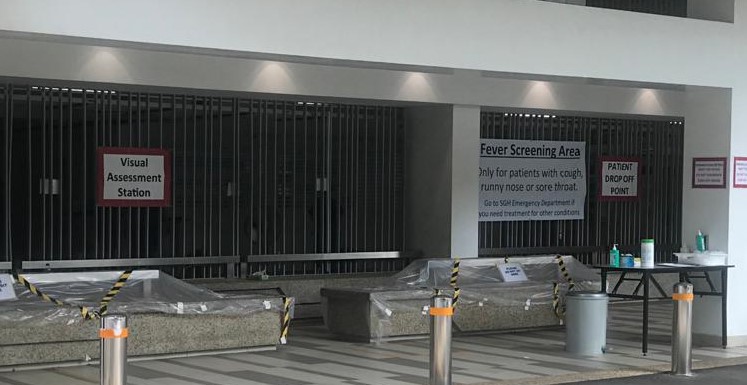


*Fig 9.* Chairs placed 1 meter apart for patients waiting to be seen .

*Fig 8.* Visual assessment station (VAS).


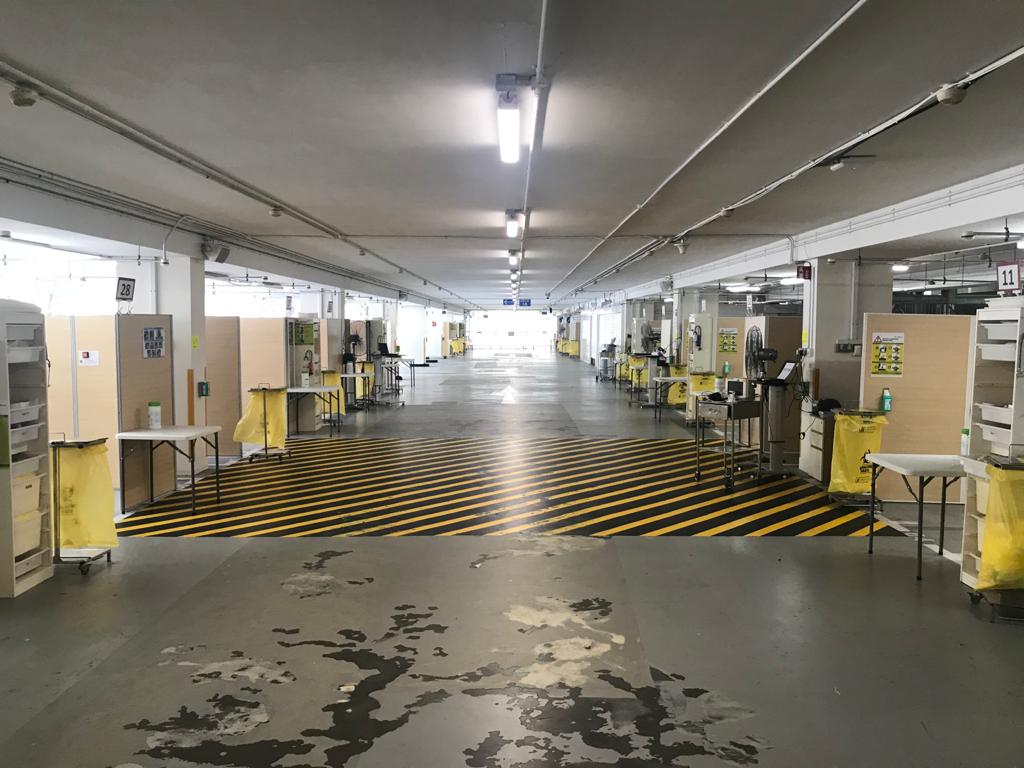

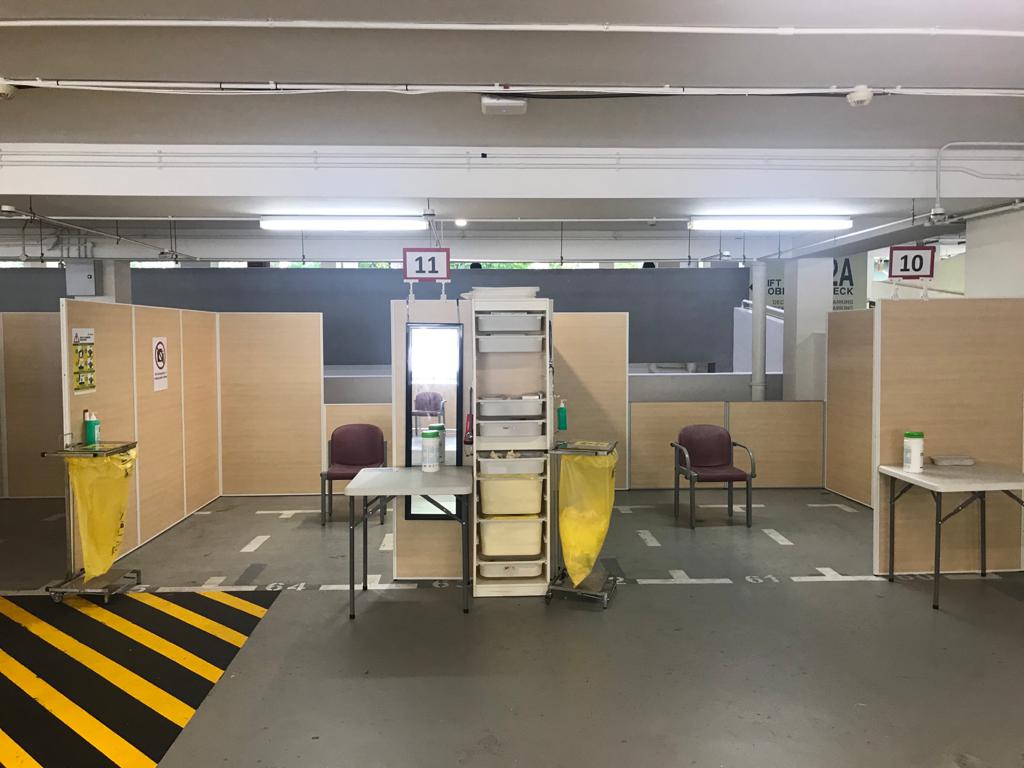


*Fig 5.* Cubicles replete with chairs, IT equipment, PPE, disinfectants, and waste disposal.


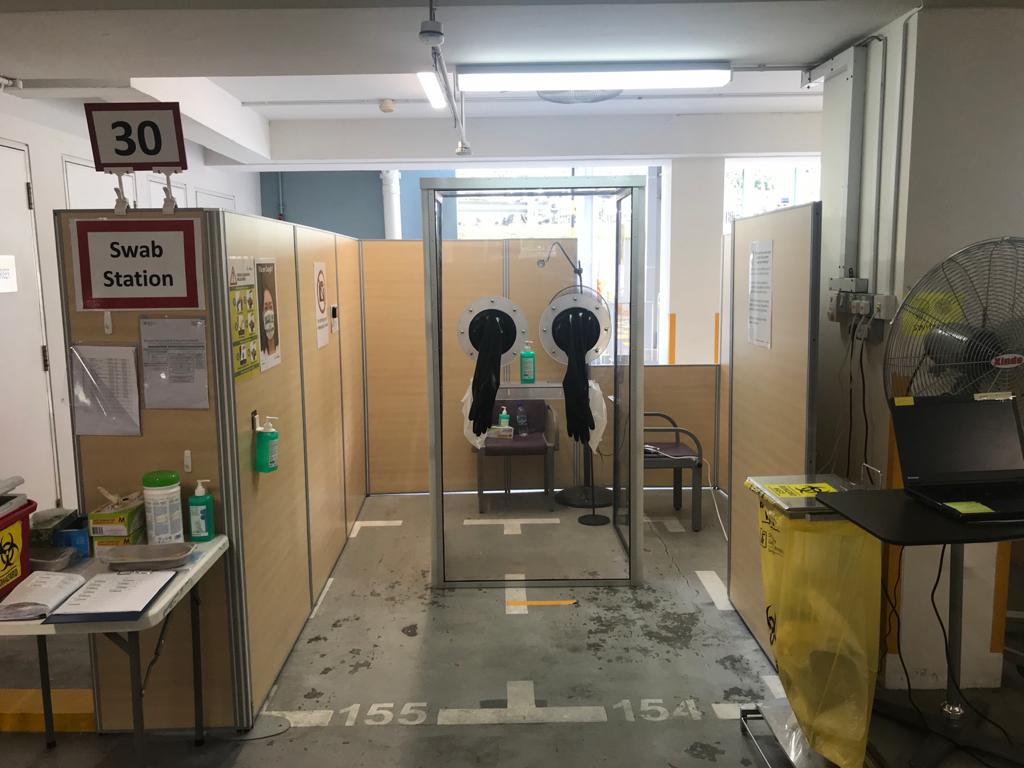

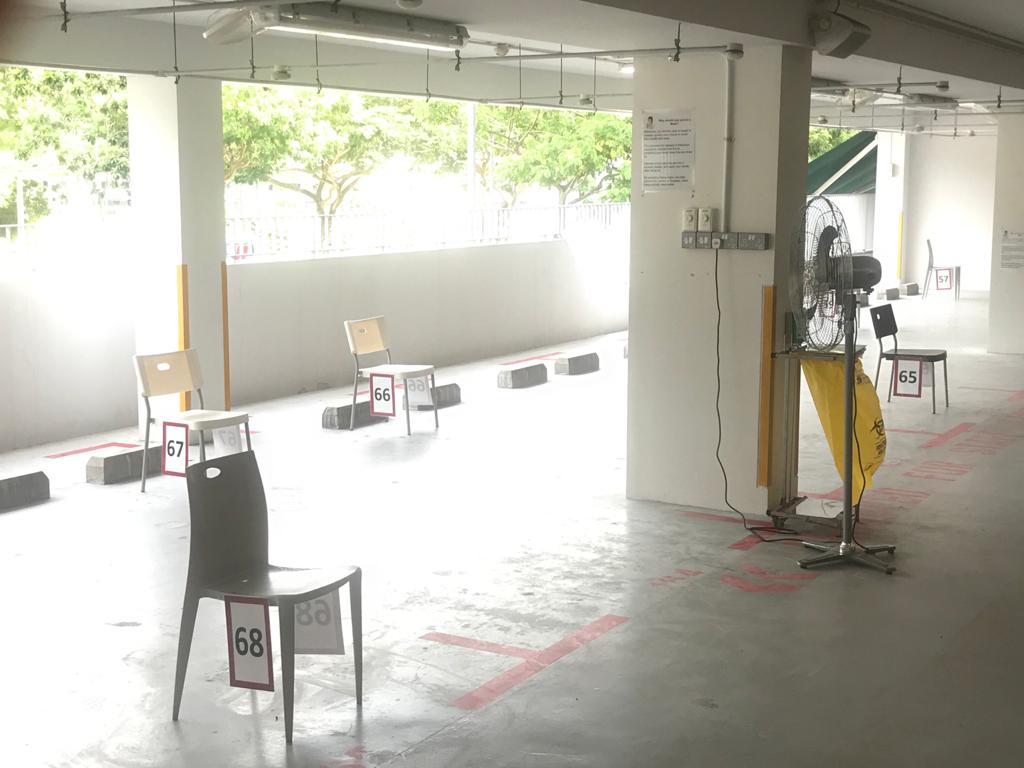


*Fig 10.* Waiting areas for patients.

*Fig 2*. Swab booth.


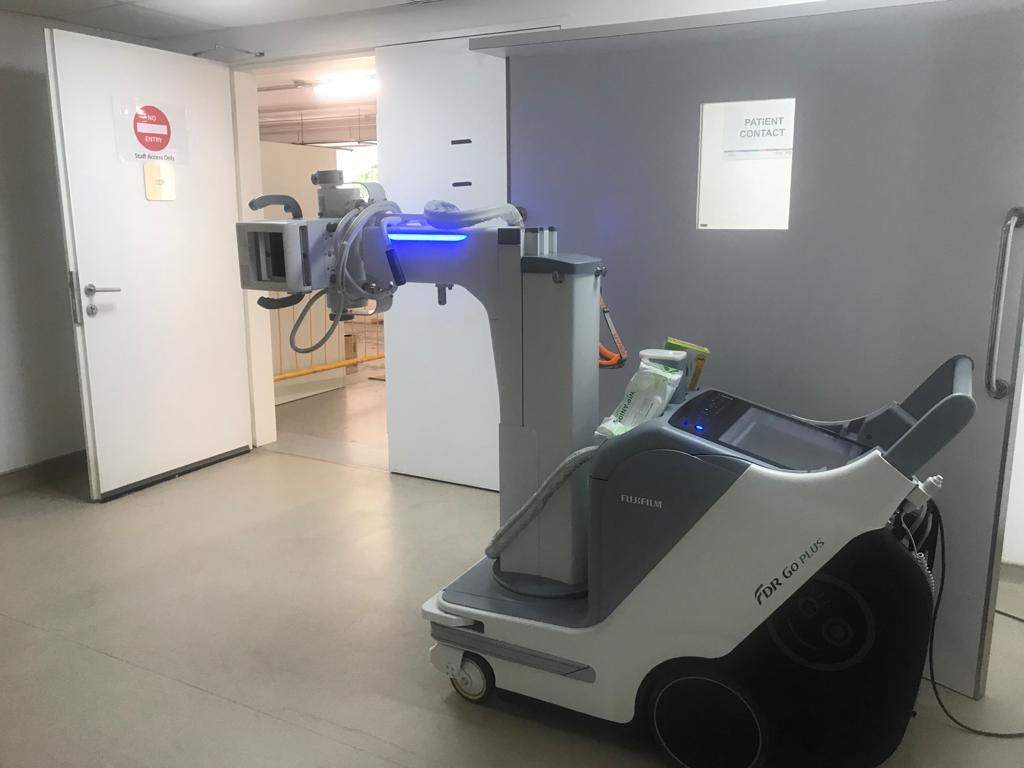

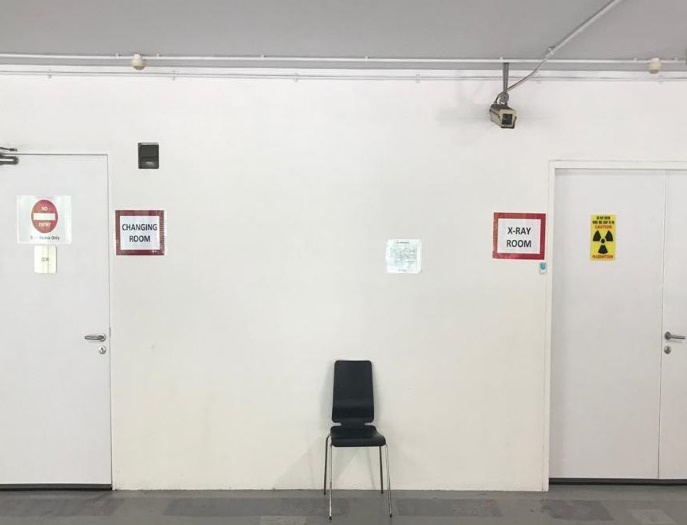


*Fig 1.* Radiology facilities in the flu screening area (FSA).


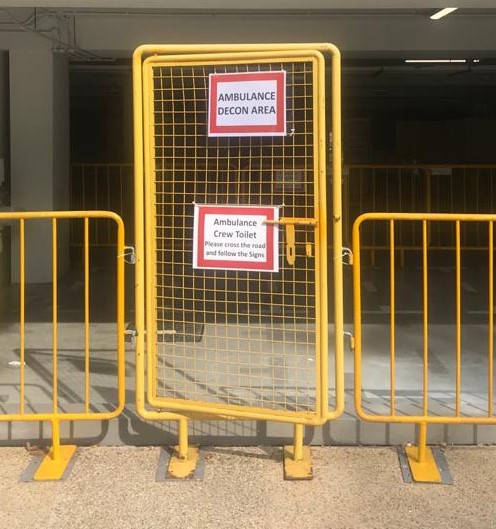

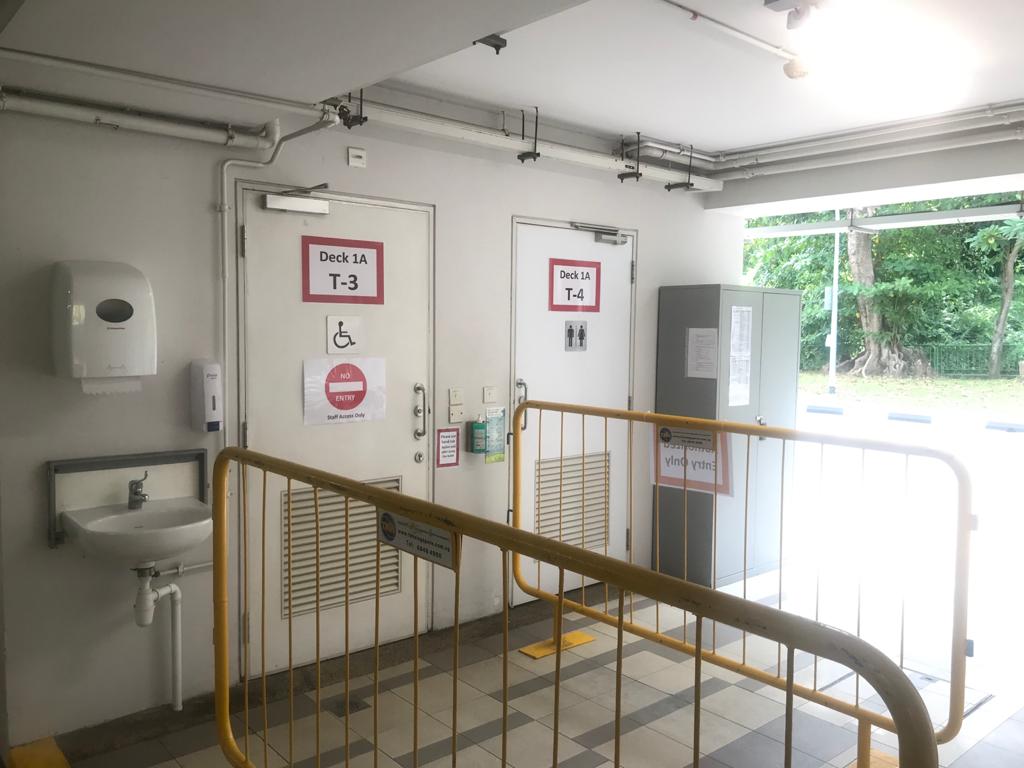


*Fig 11.* Ambulance crew decontamination areas. outside the FSA


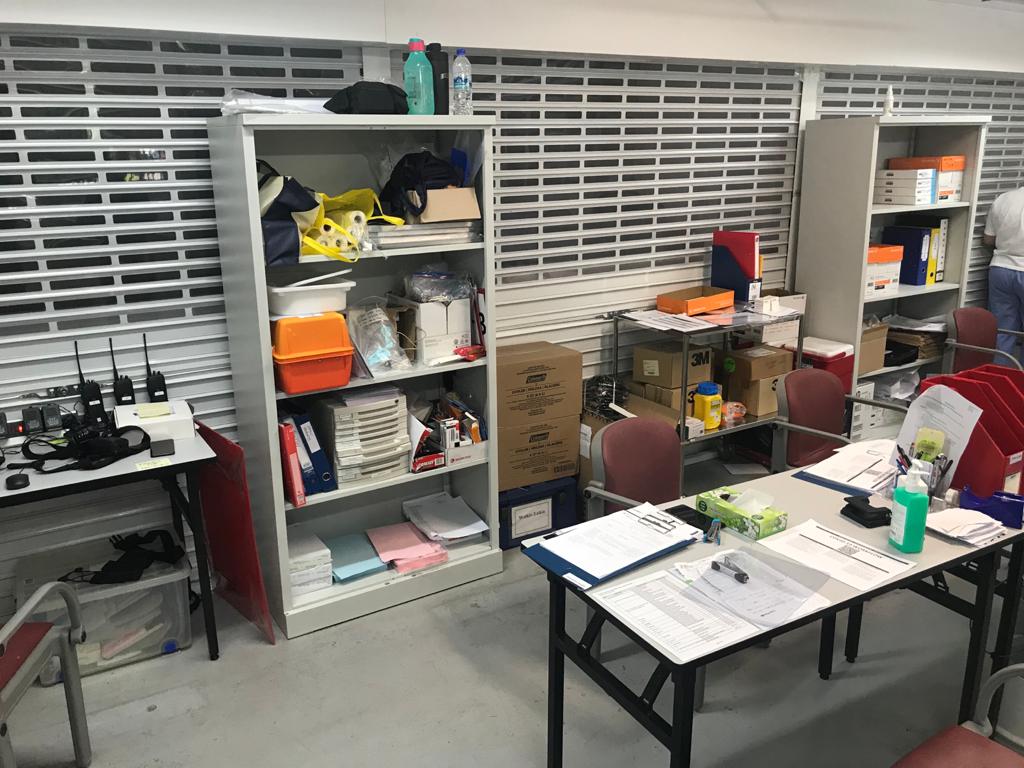

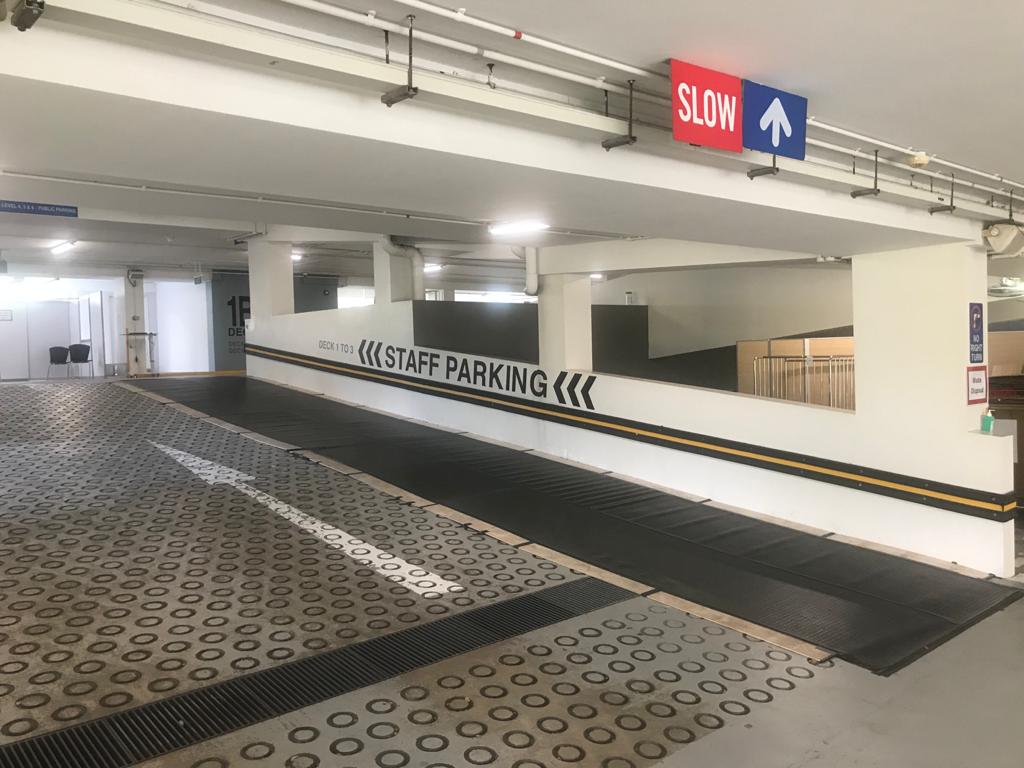


*Fig 6.* Air-conditioned, shuttered staff clinical work areas

*Fig 7.* Modified ramp

**Appendix**


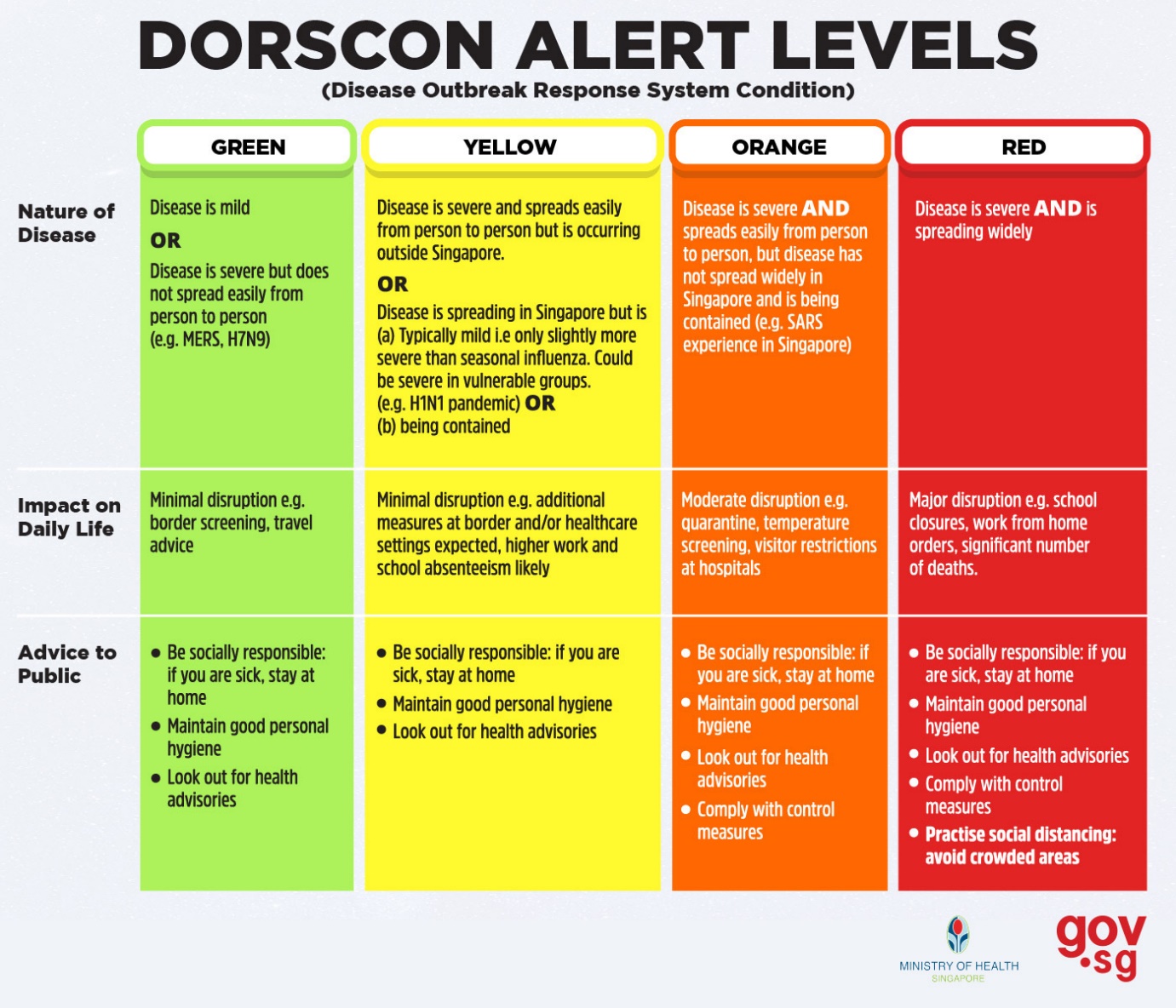
Source: <https://www.gov.sg/article/what-do-the-different-dorscon-levels-mean>
